# Supplementary material for: The root enrichment of bacteria is consistent across different stress-resistant plant species
Source: PeerJ. 2023 Jan 17;11:e14683. doi: 10.7717/peerj.14683 (PMC9854377; doi:10.7717/peerj.14683)
Supplement: Supplemental Information 1 [file peerj-11-14683-s001.docx]

Supplementary Table S1 The recorded functions of major plant-associated bacteria genera.

| **Bacteria genus** | **Function** | **Function mechanism** | **Reference** |
| --- | --- | --- | --- |
| Azospirillum | Abiotic stress tolerance | accumulate trehalose and raffinose that improve cell osmotic regulation and plasma membrane integrity | Acuña‐Rodríguez et al. 2020 |
|  | Growth promotion | nitrogen fixation to increase root nitrogen availability, keep plant hormone balance by participating in the production of IAA, gibberrelin, ethylene | Lemanceau et al. 2017 |
| Bacillus | Growth promotion | nitrogen fixation, phosphate solubilization, and phytohormone production | Miljaković et al. 2020 |
|  | Disease suppression | excrete antibiotics, cell wall hydrolases, and siderophores trigger induced systemic resistance |  |
| Bradyrhizobium | Growth promotion | root-nodulating and increase root nitrogen availability, keep plant hormone balance by participating in the production of IAA | Franche et al. 2009 |
| Burkholderia | Abiotic stress tolerance | accumulate trehalose and raffinose that improve cell osmotic regulation and plasma membrane integrity | Acuña‐Rodríguez et al. 2020 |
|  | Disease suppression | synthesize phenazines to inhibit pathogen growth | Bulgarelli et al. 2013 |
|  | Growth promotion | nitrogen fixation to increase root nitrogen availability | Franche et al. 2009 |
| Frankia | Growth promotion | fix nitrogen for a broader spectrum of plants | Franche et al. 2009 |
| Massilia | Growth promotion | correlate to plant development | Ofek et al. 2012 |
| Nitrospira | Growth promotion | nitrification to increase plant nitrogen availablility | Stein and Klotz 2016 |
| Paenibacillus | Disease suppression | modulate host defense hormones against Botrytis cinerea | Kim et al. 2017 |
|  | Growth promotion | produce IAA (indole-3-acetic acid), siderophore, and glucanase |  |
| Pantoea | Abiotic stress tolerance | degrade herbicides and other soil toxic substances | Walterson and Stavrinides 2015 |
|  | Disease suppression | produce antibiotic substances |  |
|  | Growth promotion | produce IAA, some isolates possess nitrogen fixation capabilities |  |
| Pseudomonas | Abiotic stress tolerance | accumulate trehalose and raffinose that improve cell osmotic regulation and plasma membrane integrity regulate root K/Na ion homeostasis to promote salinity tolerance | Acuña‐Rodríguez et al. 2020 |
|  | Disease suppression | synthesize coronatine, salicylic acid, and other antibiotic substances to increase plant defence and inhibit pathogen growth trigger induced systemic resistance | Lemanceau et al. 2017 |
|  | Growth promotion | keep plant hormone balance by participating in the production of IAA, cytokinin phosphorus solubilization to increase phosphorus availability | Bulgarelli et al. 2013 |
| Rhizobium | Abiotic stress tolerance | produce trehalose and polysaccharides to alleviate plant drought stress | Lemanceau et al. 2017 |
|  | Growth promotion | form root-nodulating and increase nitrogen availability, keep plant hormone balance by participating in the production of IAA, gibberrelin, acetoine |  |
| Sphingomonas | Disease suppression | reduce the growth of foliar pathogen |  |
| Stenotrophomonas | Abiotic stress tolerance | degrade and resist to natural and man-made pollutants, compounds, heavy metals | Ryan et al. 2009 |
|  | Disease suppression | produce antimicrobial compounds |  |
|  | Growth promotion | increase plant growth by hormone production, nitrogen fixation, sulphur oxidation |  |
| Streptomyces | Abiotic stress tolerance | produce phytohormones | Yangdigeri et al. 2012 |
|  | Disease suppression | synthesize phenazines to inhibit pathogen growth | Bulgarelli et al. 2013 |
| Thaumarchaeota | Growth promotion | nitrification to increase plant nitrogen availablility | Stein and Klotz 2016 |

**Reference:**

**Acuña‐Rodríguez IS, Newsham KK, Gundel PE, Torres-Díaz C, Molina-Montenegro MA. 2020.** Functional roles of microbial symbionts in plant cold tolerance. *Ecology Letters* **23(6)**:1034–1048. http://dx.doi.org/10.1111/ele.13502.

**Bulgarelli D, Schlaeppi K, Spaepen S, Van Themaat EVL, Schulze-Lefert P. 2013.** Structure and functions of the bacterial microbiota of plants. *Annual review of plant biology* **64**:807–838. http://dx.doi.org/10.1146/annurev-arplant-050312-120106.

**Franche C, Lindström K, Elmerich C. 2009.** Nitrogen-fixing bacteria associated with leguminous and non-leguminous plants. *Plant and Soil* **321(1)**:35–59. https://doi/1007/s11104-008-9833-8.

**Kim AY, Shahzad R, Kang SM, Khan AL, Lee S, Park YG, Lee WH, Lee IJ. 2017.** *Paenibacillus terrae* AY-38 resistance against *Botrytiscinerea* in *Solanumlycopersicum* L. plants through defence hormones regulation. *Journal of Plant Interactions* **12(1)**:244–253. https://doi/ 10.1080/17429145.2017.1319502.

**Lemanceau P, Blouin M, Muller D, Moënne-Loccoz Y. 2017.** Let the core microbiota be functional. *Trends in Plant Science* **22(7)**:583–595. http://dx.doi.org/10.1016/j.tplants.2017.04.008.

**Miljaković D, Marinković J, Balešević-Tubić S. 2020.** The significance of *Bacillus* spp. in disease suppression and growth promotion of field and vegetable crops. *Microorganisms* **8(7)**:1037. https://doi:10.3390/microorganisms8071037.

**Ofek M, Hadar Y, Minz D. 2012.** Ecology of root colonizing *Massilia* (*Oxalobacteraceae*). *PLoS ONE* **7(7)**:e40117. http://doi:10.1371/journal.pone.0040117.

**Ryan RP, Monchy S, Cardinale M, Taghavi S, Crossman L, Avison MB, Dow JM. 2009.** The versatility and adaptation of bacteria from the genus *Stenotrophomonas*. *Nature Reviews Microbiology* **7(7)**:514–525. https://doi.org/10.1038/nrmicro2163.

**Stein LY, Klotz MG. 2016.** The nitrogen cycle. *Current Biology* **26(3)**:R94–R98.https://doi.org/10.1016/j.cub.2015.12.021.

**Walterson AM, Stavrinides J. 2015.** *Pantoea*: insights into a highly versatile and diverse genus within the *Enterobacteriaceae*. *FEMS Microbiology Reviews* **39(6)**:968–984. https://doi:10.1093/femsre/fuv027.

**Yandigeri MS, Meena KK, Singh D, Malviya N, Singh DP, Solanki MK, Yadav AK, Arora DK. 2012.** Drought-tolerant endophytic actinobacteria promote growth of wheat (*Triticum aestivum*) under water stress conditions. Plant Growth & Regulation **68**:411–420. https://doi.org/10.1007/s10725-012-9730-2.
